# Laminin degradation by matrix metalloproteinase 9 promotes ketamine‐induced neuronal apoptosis in the early developing rat retina

**DOI:** 10.1111/cns.13428

**Published:** 2020-06-20

**Authors:** Lei Wu, Kan Zhang, Liping Sun, Jie Bai, Mazhong Zhang, Jijian Zheng

**Affiliations:** ^1^ Department of Anesthesiology Shanghai Children’s Medical Center Affiliated to School of Medicine Shanghai Jiao Tong University Shanghai China

**Keywords:** apoptosis, ketamine, laminin, matrix metalloproteinase, Zn^2+^

## Abstract

**Aims:**

During early development, laminin degradation contributes to the death of neurons. This study aims to investigate the role and regulation of laminin in ketamine‐induced apoptosis.

**Methods:**

We performed terminal deoxynucleotidyl transferase biotin‐dUTP nick end labeling (TUNEL) and immunohistochemical assays to investigate the roles of the non‐integrin laminin receptor, matrix metalloproteinase 9 (MMP9) in ketamine‐induced neuronal apoptosis. In situ zymography, Western blot, and immunofluorescence were used to explore the relationships between laminin, MMP9 activity, and Zn^2+^. Experiments were performed using whole‐mount retinas dissected from Sprague Dawley rats.

**Results:**

The TUNEL and immunohistochemical assays indicated that ketamine‐induced neuronal apoptosis in early developing rat retina. Blockade of non‐integrin laminin receptor promoted ketamine‐induced apoptosis, while non‐integrin laminin receptor activation attenuated ketamine‐induced apoptosis. Ketamine‐induced laminin degradation, possibly by enhancing the activity of MMP9. MMP9 inhibition reduced ketamine‐induced apoptosis by reducing laminin degradation. Downregulation of Zn^2+^ attenuated the increased MMP9 activity, laminin degradation caused by ketamine and significantly reduced ketamine‐induced neuronal apoptosis.

**Conclusion:**

Laminin degradation by MMP9 promoted ketamine‐induced neuronal apoptosis in early developing rat retina. The non‐integrin laminin receptor may be a pathway involved in ketamine‐induced apoptosis. Zn^2+^ downregulation may play a protective role against ketamine‐induced neuronal apoptosis through inhibiting MMP9 activity.

## 1 INTRODUCTION

Although the risk of learning and memory impairments associated with general anesthesia in children is still under debate, parents and the general public are very concerned about the safety and long‐term outcome of children undergoing general anesthesia.^1^, ^2^ Furthermore, various animal studies have demonstrated that long‐term or repeated exposure to general anesthetics can cause neuronal apoptosis in early stages of life and learning and memory impairments later in life.^3^, ^4^, ^5^ General anesthesia‐induced neurotoxicity in animals mainly occurs during the period of peak synaptogenesis, referred to as the window of vulnerability.^6^, ^7^ Although extensive studies have been carried out for the past 20 years, the mechanisms underlying general anesthesia‐induced developmental neurotoxicity still need to be elucidated, and no effective prevention or treatment strategies have been developed thus far.^7^, ^8^

General anesthetics promote central nervous system inhibition via activation of the gamma‐aminobutyric acid type A receptor (GABAA‐R) and/or blockade of the N‐methyl‐D‐aspartic acid receptor (NMDAR).^9^ During early neuronal development, both GABAA‐R activation and long‐term NMDAR blockade can cause intracellular calcium disturbances and neuronal apoptosis.^8^, ^10^ In addition to these effects caused by the receptors, general anesthesia may also induce neuronal apoptosis by influencing the extracellular environment including brain‐derived neurotrophic factor, inflammatory mediators, cell‐extracellular matrix (ECM), etc^11^, ^12^, ^13^ During early postnatal development, the ECM plays crucial roles in proliferation, migration, and differentiation of neural cells and form loose ECM fiber‐like structures, which occurs in parallel with synaptic development.^14^ The ECM consists of proteins including collagen, fibronectin, and laminin.^14^ Laminins are indispensable building blocks of cellular networks and of extracellular polymers, which determine the architecture and physiology of basement membranes.^15^ Previous studies have demonstrated that laminin degradation can lead to the death of developing neurons by affecting events downstream of protein kinase B (Akt) activation.^16^ Plasmin‐mediated laminin degradation is critical for ethanol‐induced neuronal apoptosis.^17^ These studies indicate that laminin may be a pathway leading to the apoptosis of developing neurons. However, whether laminin degradation contributes to the neuronal apoptosis induced by ketamine, a NMDAR antagonist, remains to be elucidated.

The effects of laminins are often mediated through interactions with integrin and non‐integrin laminin receptors (LR).^18^ The non‐integrin LR was the first identified laminin receptor.^19^ Interactions between the non‐integrin LR and laminin play a key role in mediating changes in ECM that affect cell adhesion, neurite outgrowth, angiogenesis, and apoptosis.^20^, ^21^ The non‐integrin LR is required for maintenance of cell viability by preventing apoptosis.^22^, ^23^ Previous study demonstrated that siRNA‐mediated knockdown of non‐integrin LR reduced FAK phosphorylation, leading to cell apoptosis.^24^ However, whether non‐integrin LR involved in ketamine‐induced neuronal apoptosis needs to be elucidated.

Laminin can be targeted and proteolytically cleaved to regulate neuronal function by active matrix metalloproteinases (MMPs), which widely exist in the early developmental period.^25^, ^26^, ^27^ A previous study demonstrated that MMP9 can regulate neuronal survival by degrading laminin and modulating the laminin‐integrin β1 signaling pathway.^16^, ^28^ Moreover, as a zinc‐dependent endopeptidase, the activity of MMP9 is closely related to the concentration of free zinc ions. Zn^2+^ downregulation has been demonstrated to contribute to the inhibition of MMP activity.^29^, ^30^ Nevertheless, it remains unclear whether altering MMP9 activity by up‐ or downregulating Zn^2+^ affects ketamine‐induced neuronal apoptosis in early developing rat. We therefore speculated that altering MMP9 activity by up‐ or downregulating Zn^2+^ may affect laminin degradation and ketamine‐induced neuronal apoptosis. In the present study, we explored the possible effects of laminin and MMP9 on ketamine‐induced neuronal apoptosis in the developing rat retina.

## 2 METHODS

### 2.1 Animals

All experimental procedures were approved by the Animal Care Committee of Shanghai Children's Medical Center, Shanghai Jiao Tong University School of Medicine. All experiments strictly adhered to the ARVO Statement for the Use of Animals in Ophthalmic and Vision Research and followed the Guidelines for the Care and Use of Laboratory Animals published by the US National Institutes of Health (National Institutes of Health Publication No. 85‐23, revised in 1996). Sprague Dawley rats aged 7 postnatal days (P7) were provided by the Experimental Animal Center of Shanghai Children's Medical Center. Male and female rats were both used in our study. All rat pups were housed with their mothers under a 12‐hour light/dark cycle.

### 2.2 Tissue dissection

Retinal tissues were prepared as previously described.^6^ Briefly, the eyeballs of P7rats were rapidly removed with scissors following instantaneous decapitation. The extracted eyeballs were then further dissected in an ice‐cold bath of artificial cerebrospinal fluid (ACSF) composed of the following (in mmol/L): 119 NaCl, 26.2 NaHCO_3_, 2.5 KCl, 1.3 MgCl_2_, 11 D‐glucose, 1.0 KH_2_PO_4_ and 2.5 CaCl_2_ equilibrated with 95% O_2_ and 5% CO_2_. To facilitate full exposure of the retina to drugs, an incision spanning approximately 1/5th of the circumference of the eyeball was made between the edges of the cornea and sclera. Following 1 hour of recovery in ACSF bubbled with a mixture of 95% O_2_/5% CO_2_ gas at 37°C, the eyeballs were incubated with ketamine, antagonists or agonists (either in combination or separately) in ACSF bubbled with a mixture of 95% O_2_/5% CO_2_ gas at 37°C for 5 hours.

### 2.3 Drugs and chemicals

Normal ACSF was replaced by ACSF with or without drugs depending on the experimental design. The following drugs were used: ketamine (Gutian Pharmaceutical Company), MMP9 inhibitor (Calbiochem), and N, N, N’, N’‐tetrakis (2‐pyridylmethyl) ethylenediamine (TPEN) (Med Chem Express Company), ZnCl_2_ (Gutian Pharmaceutical Company), the non‐integrin laminin receptor (LR) agonist Laminin (925‐933) (Adooq Bioscience Company) and the LR antagonist NSC47924 (Yifei Biotechnology Company). All drugs were dissolved in ACSF except for NSC47924 and TPEN, which were first dissolved as a stock solution in DMSO, and then diluted to ACSF with a DMSO concentration <0.1%.

### 2.4 Immunohistochemistry

Immunohistochemistry was performed according to experimental methods described previously.^6^, ^9^ After drug treatment, the retinas were dissected from the eyeballs in an ice‐cold bath of ACSF and fixed in 4% paraformaldehyde for 24 hours. The fixed retinas were then incubated with ethanol and xylene, after which they were infiltrated with paraffin. The paraffin‐embedded retinas were cut into 4‐6 μm‐thick slices using a microtome (Leica‐2135, Leica). After endogenous peroxidase inactivation and heat‐induced antigen retrieval, the tissue sections were first incubated with a primary antibody against cleaved caspase‐3 (AC3; Table 1) overnight at 4℃. The sections were then incubated with a horseradish peroxidase‐conjugated goat anti‐rabbit immunoglobulin G (IgG) secondary antibody (PV‐9001, ZSGB‐BIO) at 37°C for 1 hour. Anti‐AC3 immunoreactivity was detected using 3,30‐ diaminobenzidine (ZLI‐9017, ZSGB‐BIO) oxidization. AC3‐positive neurons (brown staining cells) were then examined using a light microscope.

**Table 1:** List of antibodies used in the immunohistochemical experiments

Antibody	
Cleaved caspase‐3	Cell Signaling Technology Cleaved Caspase‐3 (Asp175) Antibody #9661
Cleaved MMP9	Cell Signaling Technology MMP9 Antibody #3852
MMP9	Abcam; Rabbit polyclonal (anti)‐MMP9 antibody (ab38898)
Laminin	Abcam; Rabbit polyclonal (anti)‐Laminin (ab11575)
GAPDH	Abcam; Rabbit monoclonal [EPR16891] to GAPDH (ab181602)

Abbreviation: MMP9: matrix metalloproteinase 9.

For immunofluorescence, the retinal sections were first incubated with a primary antibody against laminin or MMP9 (Table 1) overnight at 4°C. They were then incubated with an Alexa Fluor 594‐conjugated goat anti‐rabbit IgG secondary antibody (Thermo Scientific) for 30 minutes, followed by DAPI for 5 minutes. Images were captured using a fluorescence microscope (Leica TCS SP8; Leica). Each image was composed of 1384 × 1040 pixels and had a resolution of 150 pixels/inch. Each group comprised five retina samples. Five discontinuous images randomly obtained using a fluorescence microscope were analyzed in each sample. To compare intensities between samples, the same exposure time was used for all samples. Image Pro Plus 6.0 (Media Cybernetics Company) was used to determine fluorescence intensity.

### 2.5 Terminal deoxynucleotidyl transferase biotin‐dUTP nick end labeling assay

For the TUNEL assay, retinal tissue sections were dewaxed, hydrated, and treated with proteinase K for 30 minutes according to the manufacturer's instructions (TUNEL AP kit, Roche Applied Science). The sections were initially incubated with 3% hydrogen peroxide for 15 minutes. Following this, the sections were then incubated with a terminal deoxynucleotidyl transferase reaction mix for 60 minutes at 37°C, followed by DAPI for 5 minutes. TUNEL‐positive cells (red staining cells) were observed via fluorescence microscopy. The numbers of AC3‐positive cells and TUNEL‐positive cells in randomly selected image areas were counted in a double‐blinded manner. Each group comprised five retina samples. Five discontinuous images randomly obtained using a light microscope or fluorescence microscope (400 × magnification) were analyzed to determine the numbers of AC3‐positive and TUNEL‐positive cells in each retina. Image Pro Plus 6.0 (Media Cybernetics Company) was used to determine the number of apoptotic cells in the ganglion cell layer (GCL). The percentage of apoptotic cells was calculated using the following formula: the number of apoptotic cells/the total number of cells in the GCL.

### 2.6 Western blot assay

The protein concentration of retinal extracts and cell lysates was determined using a BCA kit (Beyotime Biotechnology), and 40 μg of proteins was electrophoresed in 10% SDS‐PAGE. The separated proteins were then transferred to nitrocellulose membranes (Millipore). Bovine serum albumin in Tris buffer saline was used to block nonspecific binding. The membranes were incubated with a primary antibody against MMP9 at 4°C overnight. GAPDH was used as a loading control (Table 1). The membranes were then incubated with a horseradish peroxidase‐streptavidin‐conjugated secondary antibody for 1 hour at room temperature. Antibody detection was performed via enhanced chemiluminescence (Thermo Fisher Scientific), and the intensity of the bands was quantified by densitometric analysis using Gel Pro Analyzer software (Media Cybernetics, Inc).

### 2.7 In situ gelatin substrate zymography

Fluorescent in situ gelatin substrate zymography was used to localize MMP9 proteolytic activity according to the manufacturer's instructions (Genmed, GMS80062.1). Gel‐embedded retinas were cut into 8‐10 μm‐thick slices using a freezing microtome (Leica CM1950; Leica). Reagent B was warmed to room temperature in the dark, and Reagent A was heated in a microwave. Following this, 400 mL Reagent A was placed in a 1.5 mL centrifuge tube and incubated for 10 minutes at 37°C. Then, 50 mL reagent B preheated at 37°C was added to reagent A. Subsequently, 40 mL of this mixed liquid was immediately added to each unfixed frozen sample. The samples were then covered with coverslips and incubated in a 4°C refrigerator for 10 minutes until the colloid coagulated. Finally, the tissue samples were incubated in a 37°C incubator for 1 hour. The fluorescence intensity of the samples was observed via fluorescence microscopy (Leica TCS SP8; Leica). The same exposure time was used for all samples. Image Pro Plus 6.0 (Media Cybernetics Company) was used to determine fluorescence intensity. Fluorescent of MMP9 activity was presented as the percentage of fluorescent intensity in control group.

### 2.8 Statistical analysis

Data are expressed as mean ± standard deviation. All statistical data were analyzed using GraphPad Prism 5 software (GraphPad Software Inc) or IBM SPSS Statistics 23 (SPSS Inc, IBM Corporation). The Shapiro‐Wilk test was used to assess the normality of the data distribution. Student's *t* test was used to analyze comparisons of normally distributed data. Multiple comparisons were performed using one‐way analysis of variance followed by the least significant difference post hoc test. Data that do not exhibit a normal distribution were analyzed using the Mann‐Whitney or Kruskal‐Wallis test. *P* values < .05 were considered statistically significant.

## 3 RESULTS

### 3.1 Laminin is involved in ketamine‐induced neuronal apoptosis in developing rat retina

**Figure 1:**
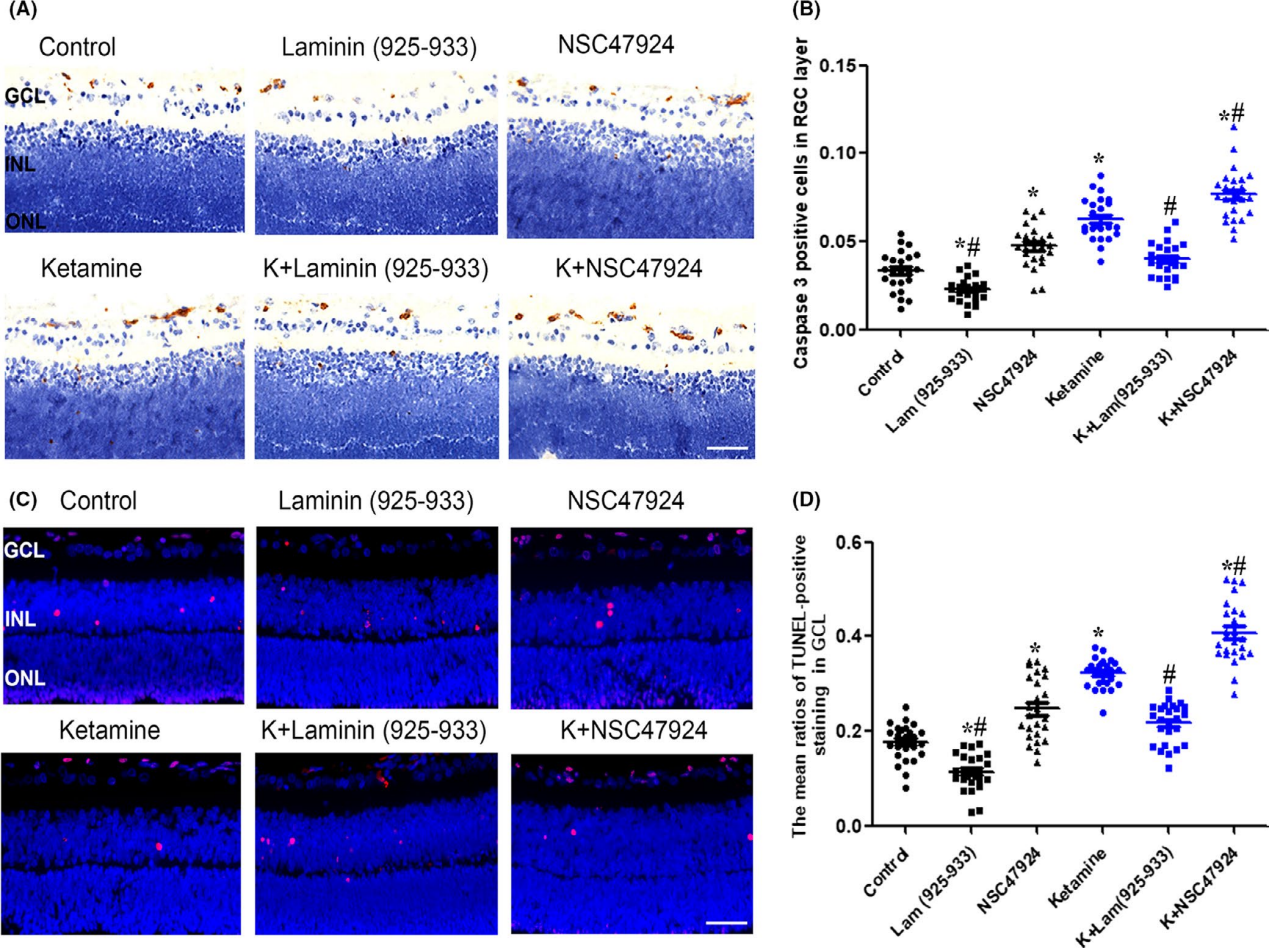
Laminin is involved in ketamine‐induced neuronal apoptosis in the GCL of the rat retina at P7. A, Representative photomicrograph of caspase‐3‐positive staining (brown) in the GCL, INL, and ONL (scale bar = 25 μm). B, Ratios of caspase‐3‐positive cells in the GCL in different treatment groups. C, Representative photomicrograph of TUNEL‐positive staining (red) in the GCL (scale bar = 25 μm). D, Ratios of TUNEL‐positive cells in the GCL in different treatment groups. GCL: ganglion cell layer;INL: inner nuclear layer; ONL: outer nuclear layer; P7: postnatal day 7; TUNEL: terminal deoxynucleotidyl transferase biotin‐dUTP nick end labeling. *P < .05 relative to the control group; #*P* < .05 relative to the ketamine group

To investigate the role of laminin in physiological apoptosis, we explored the effects of the non‐integrin LR antagonist NSC47924 and agonist Laminin (925‐933) on retinal apoptosis in P7 rats. The immunohistochemistry and TUNEL assays both revealed that exposure to 100 μmol/L Laminin (925‐933) significantly decreased the extent of neuronal apoptosis in the GCL of the retina (Figure 1). The percentage of AC3‐positive neurons decreased from 3.4 ± 1.1% to 2.2 ± 0.6% (*P* = .02, n = 5) and the percentage of TUNEL‐positive cells decreased from 16.6 ± 3.3% to 10.7 ± 2.9% (*P* = .001, n = 5). Conversely, 100 μΜ NSC47924increased the ratio of neuronal apoptosis in the GCL of the rat retina. The percentage of AC3‐positive neurons increased from 3.4 ± 1.1% to 4.9 ± 1.2% (*P* < .001, n = 5) and the percentage of TUNEL‐positive cells increased from 16.6 ± 3.3% to 23.7 ± 5.1% (*P* < .05, n = 5).

We next tested whether laminin plays a role in ketamine‐induced apoptosis in the rat retina. Exposure to 150 μmol/L ketamine (5 hours) significantly increased the percentage of retinal ganglion cells expressing markers of apoptosis from 3.4 ± 1.1% to 6.3 ± 1.2% (n = 5, *P* < .001). The percentage of TUNEL‐positive cells in the GCL increased from 16.6 ± 3.3% to 32.2 ± 3.1% following ketamine treatment (*P* = .001, n = 5). The immunohistochemical and TUNEL assays revealed that exposure to 100 μmol/L Laminin (925‐933) decreased the extent of ketamine‐induced neuronal apoptosis in the GCL (Figure 1). The percentage of AC3‐positive neurons decreased from 6.3 ± 1.2% to 3.9 ± 0.9% (*P* < .001, n = 5), and the percentage of TUNEL‐positive cells decreased from 32.2 ± 3.1% to 21.3 ± 4.5% (*P* < .001, n = 5). Conversely, 100 μΜ NSC47924 increased the percentage of AC3‐positive neurons from 6.3 ± 1.2% to 7.6 ± 1.5% (*P* = .01, n = 5) and the percentage of TUNEL‐positive cells from 32.2 ± 3.1% to 41.1 ± 7.2% (*P* < .001, n = 5).

### 3.2 Ketamine decreased the expression of laminin and MMP9 inhibition attenuated ketamine‐induced decrease in laminin expression

**Figure 2:**
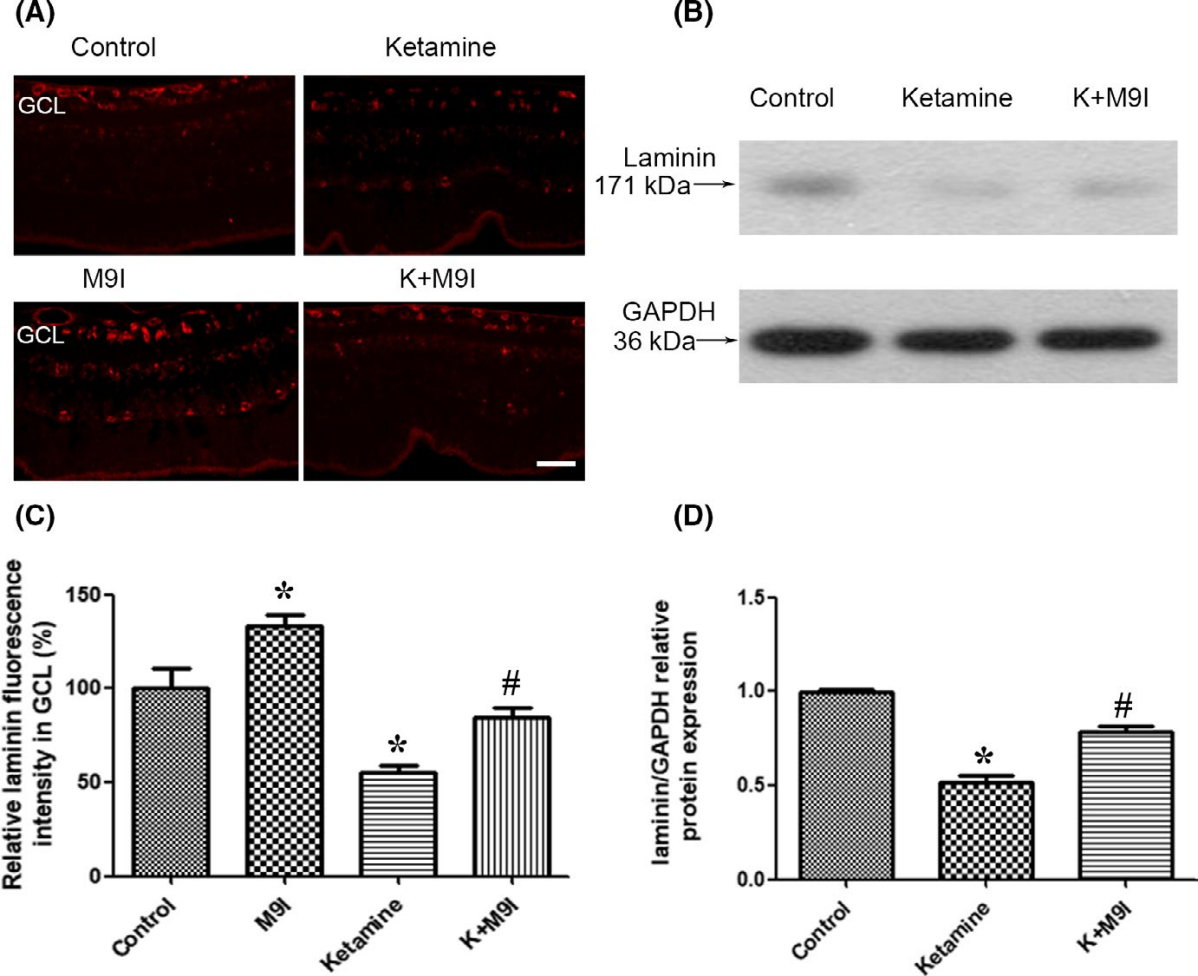
Ketamine decreased the expression of laminin and treatment with MMP9 inhibitor attenuated the ketamine‐induced decrease in laminin expression in the GCL of the rat retina at P7. A, Immunofluorescence micrographs of laminin (scale bar = 25 μm) in normal and drug‐administered retinas from P7 rats. Rat retinas were treated with ketamine, a MMP9 inhibitor, or ketamine + MMP9 inhibitor. B, Treatment with a MMP9 inhibitor attenuated ketamine‐induced laminin proteolysis demonstrated by Western blots. C, Relative laminin fluorescence intensity in the GCL (%) in each group. Fluorescent of laminin was presented as the percentage of fluorescent intensity in control group. D, Relative band intensities of laminin chain standardized against GAPDH bands in each group demonstrated by Western blots. GCL: ganglion cell layer; MMP9: matrix metalloproteinase 9; M9I: MMP9 inhibitor; P7: postnatal day 7. *P < .05 relative to the control group; #*P* < .05 relative to the ketamine group

To investigate the effects of ketamine on laminin expression, we performed immunofluorescence and Western blot experiments in the rat retina. After incubation with 150 μmol/L ketamine, the expression of laminin in the GCL of the retina significantly decreased (Figure 2A,C). To determine whether the ketamine‐induced decrease in laminin expression was related to MMP9 activity, we examined the effects of the MMP9 inhibitor (50 μmol/L) on ketamine‐induced laminin degradation. Western blots of retinal extracts and immunofluorescence both demonstrated that administration of the MMP9 inhibitor attenuated ketamine‐induced laminin degradation (Figure 2, Supplementary Figure), indicating that specific MMP9 inhibition may protect laminin from degradation.

### 3.3 MMP9 inhibition reduced ketamine‐induced neuronal apoptosis

**Figure 3:**
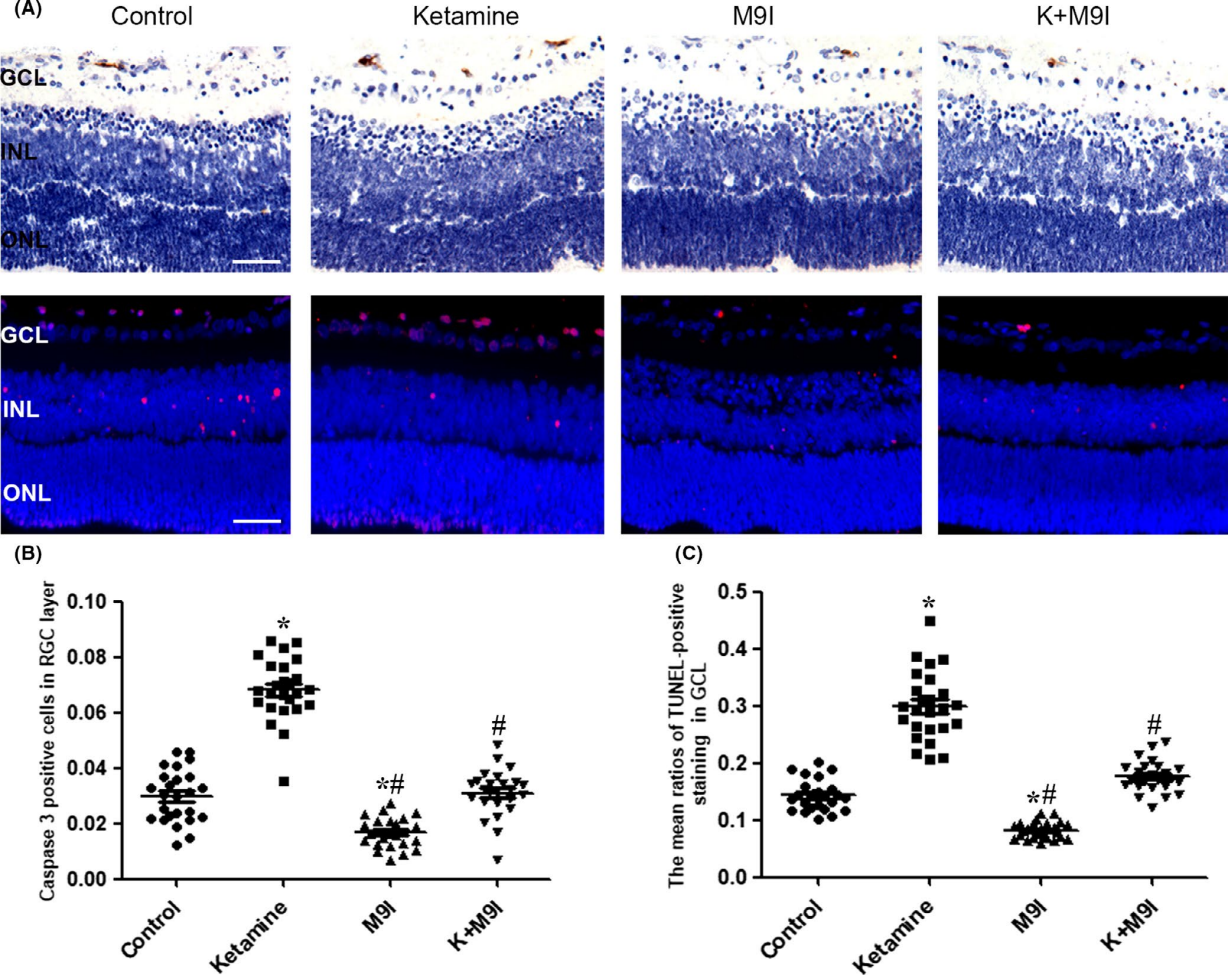
Effects of MMP9 inhibitor on ketamine‐induced neuronal apoptosis in the developing rat retina. A, Representative photomicrograph of caspase‐3‐positive staining (brown) and TUNEL‐positive staining (red) in the GCL, INL, and ONL (scale bar = 25 μm) in the ketamine and MMP9 inhibitor‐treated groups. B‐C, Dot‐plots showing the percentages of AC3‐positive cells and TUNEL‐positive cells in the GCL following treatment with a MMP9 inhibitor on physiological and ketamine‐induced neuronal apoptosis. AC3: activated cleaved caspase‐3; GCL: ganglion cell layer; INL: inner nuclear layer; ONL: outer nuclear layer; K: ketamine; MMP9: matrix metalloproteinase 9; M9I: MMP9 inhibitor; TUNEL: terminal deoxynucleotidyl transferase biotin‐dUTP nick end labeling.*P < .05 relative to the control group; #*P* < .05 relative to the ketamine group

We next explored the role of MMP9 in regulating neuronal apoptosis in P7 rats. Our immunohistochemical experiments revealed that MMP9 inhibitor (50 μmol/L) treatment significantly decreased the extent of physiological apoptosis in the GCL of the rat retina. The percentage of AC3‐positive neurons decreased from 3.0 ± 1.1% to 1.8 ± 0.7% (*P* = .013). The TUNEL assay results further verified the immunohistochemistry results. Treatment with the MMP9 inhibitor decreased the percentage of TUNEL‐positive neurons from 14.5 ± 2.8% to 8.1 ± 1.3% (*P* = .006) (Figure 3).

We further investigated whether MMP9 is involved in ketamine‐induced neuronal apoptosis. Treatment with MMP9 inhibitor significantly alleviated ketamine‐induced neuronal apoptosis at P7. The percentage of AC3‐positive neurons decreased from 6.4 ± 1.1% to 3.1 ± 0.8% (*P* < .001). Similarly, the percentage of TUNEL‐positive neurons decreased from 31.8 ± 5.9% to 18.1 ± 2.3% (*P* < .001, n = 5) after treatment with MMP9 inhibitor (Figure 3).

### 3.4 Ketamine increased the expression and activity of MMP9 in developing rat retina

**Figure 4:**
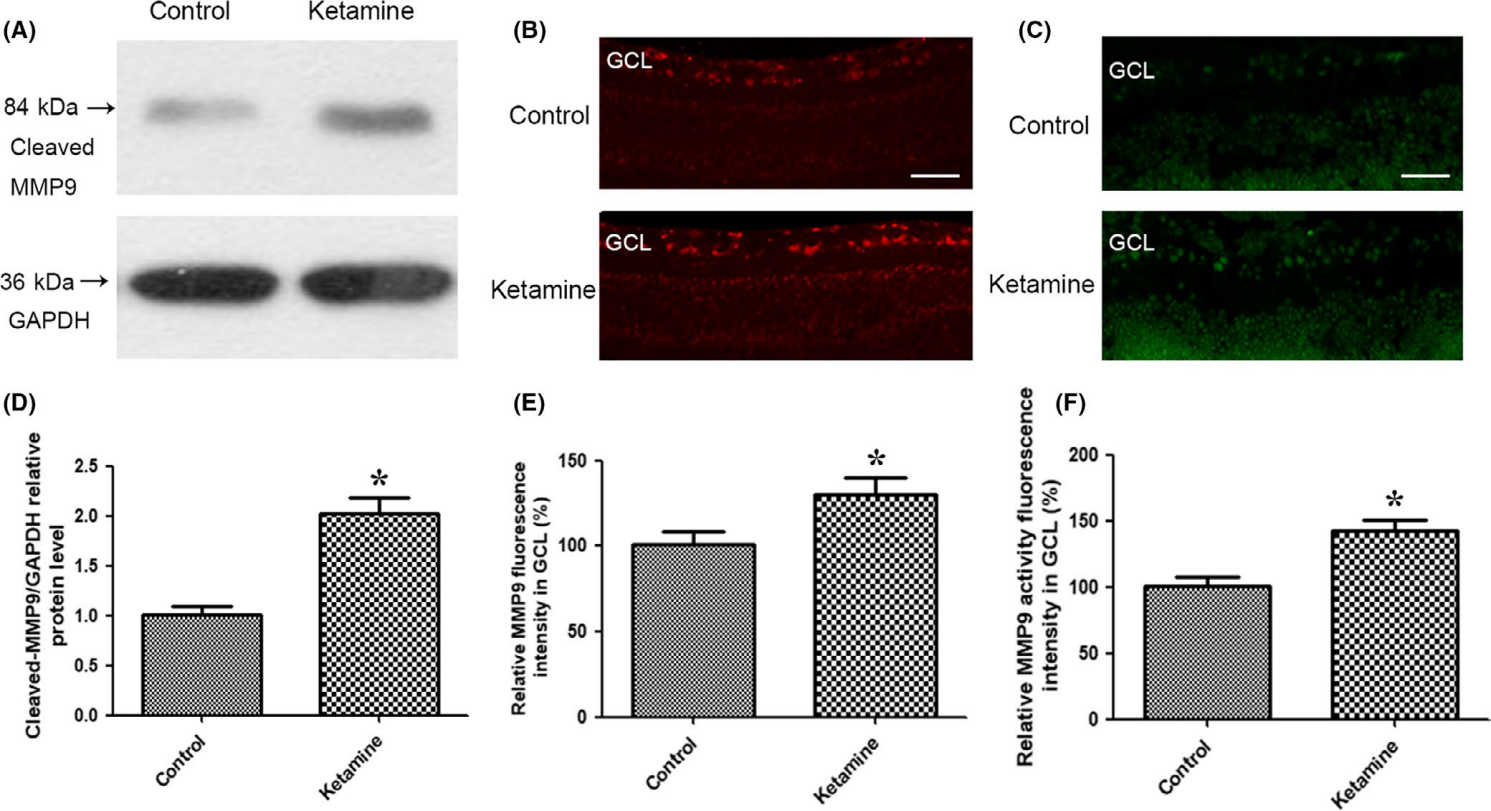
Ketamine increased the expression and activity of MMP9 in the GCL of the rat retina at P7. A, Ketamine increased MMP9 expression examined by Western blots. B, Representative immunofluorescence photomicrographs of MMP9 in normal and ketamine‐treated retinas (scale bar = 25μm). C, In situ zymography visualized under a microscope revealed an increase in MMP9 activity in RGCs following ketamine treatment (scale bar = 25 μm). D, Relative band intensities of cleaved‐MMP9 chain standardized against GAPDH bands in the control and ketamine groups examined by Western blots. E, Relative MMP9 fluorescence intensity in the GCL (%) in the control and ketamine groups. Fluorescent of MMP9 expression or activity in ketamine group was presented as the percentage of fluorescent intensity in control group. F, Relative MMP9 activity fluorescence intensity in the GCL (%) following ketamine treatment. GCL: ganglion cell layer; MMP9: matrix metalloproteinase 9; P7: postnatal day 7; RGC: retinal ganglion cell. *P < .05 relative to the control group

To detect potential changes in MMP9 after ketamine administration, the protein expression of MMP9 in the rat retina was examined by Western blot and immunofluorescence. Notably, 150 μmol/L ketamine significantly increased the expression of MMP9 in the rat retina (Figure 4A,D, Supplementary Figure). Our immunofluorescence experiments revealed that exposure to ketamine increased the expression of MMP9 in the GCL of the retina at P7 (Figure 4B,E). A marked upregulation of MMP9 activity in the GCL of the retina was detected in rat pups after ketamine treatment using in situ zymography (Figure 4C,F).

### 3.5 Downregulation of Zn^2+^ reduced the activity of MMP9 and laminin degradation in developing rat retina

**Figure 5:**
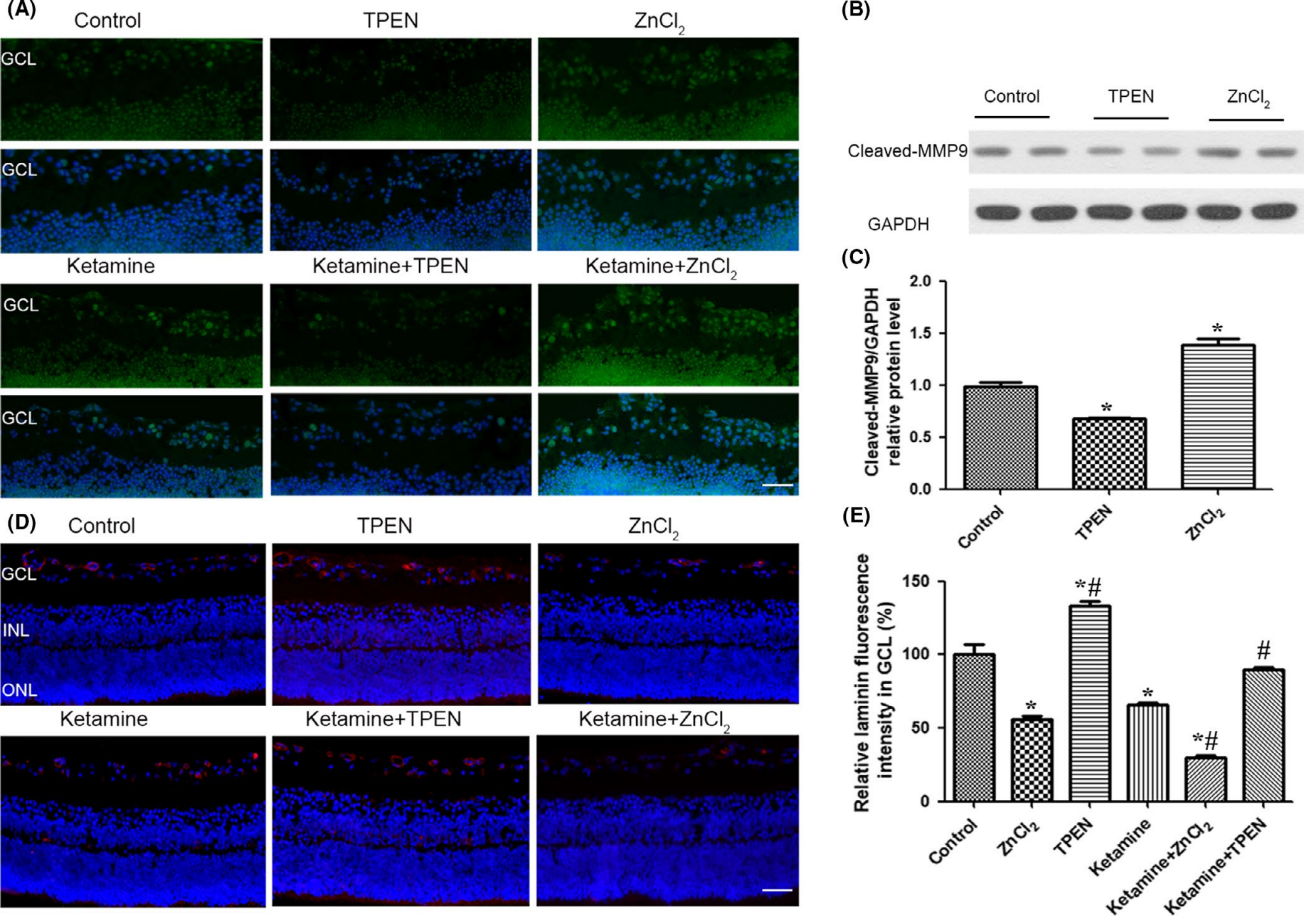
Treatment with a Zn^2+^ chelator reduced the activity of MMP9 and laminin degradation in the GCL of the rat retina at P7. A, In situ zymography visualized under a microscope revealed an increase in MMP9 activity in RGCs following ketamine and Zn^2+^ treatment and a decrease in MMP9 activity following TPEN treatment (scale bar = 25 μm). B, Western blots showed that TPEN decreased the expression of the cleaved MMP9 and zinc ions had the opposite effect. C, Relative laminin/GAPDH protein expression in different groups examined by Western blots. D, Representative photomicrograph of laminin staining (red) in the GCL following ZnCl_2_, TPEN, and ketamine treatment (scale bar = 25 μm). E, Relative laminin fluorescence intensity in the GCL (%) after the administration of drugs.GCL: ganglion cell layer; INL: inner nuclear layer; ONL: outer nuclear layer; MMP9: matrix metalloproteinase 9; P7: postnatal day 7; RGC: retinal ganglion cell; TPEN: N, N, N’, N’‐tetrakis (2‐pyridylmethyl) ethylenediamine. *P < .05 relative to the control group

To determine whether changes in the concentration of Zn^2+^ affect MMP9 activity, we performed in situ gelatin substrate zymography in the rat retina. We found that 100 μmol/L ZnCl_2_ significantly increased the gelatinolytic activity of MMP9 in the GCL of the retina. Conversely, 100 μmol/L TPEN reduced MMP9 activity. TPEN also significantly attenuated the ketamine‐induced increase in MMP9 activity in the GCL of the retina (Figure 5A). The Western blot results showed that compared to the control group, the expression of cleaved MMP9 was increased by ZnCl_2_ treatment and decreased by 100 μmol/L TPEN (Figure 5B,C, Supplementary Figure).

We further explored the influence of Zn^2+^ concentration on laminin expression in the GCL in P7 rats. The immunofluorescence experiments revealed that 100 μmol/L ZnCl_2_ significantly reduced the expression of laminin in the GCL (*P* < .05). Conversely, 100 μmol/L TPEN increased the expression of laminin compared to the control group (*P* < .05). Downregulation of Zn^2+^ by TPEN also significantly attenuated the ketamine‐induced decrease in laminin expression (Figure 5D,E).

### 3.6 Downregulation of Zn^2+^reduces ketamine‐induced neuronal apoptosis in early developing rat retina

**Figure 6:**
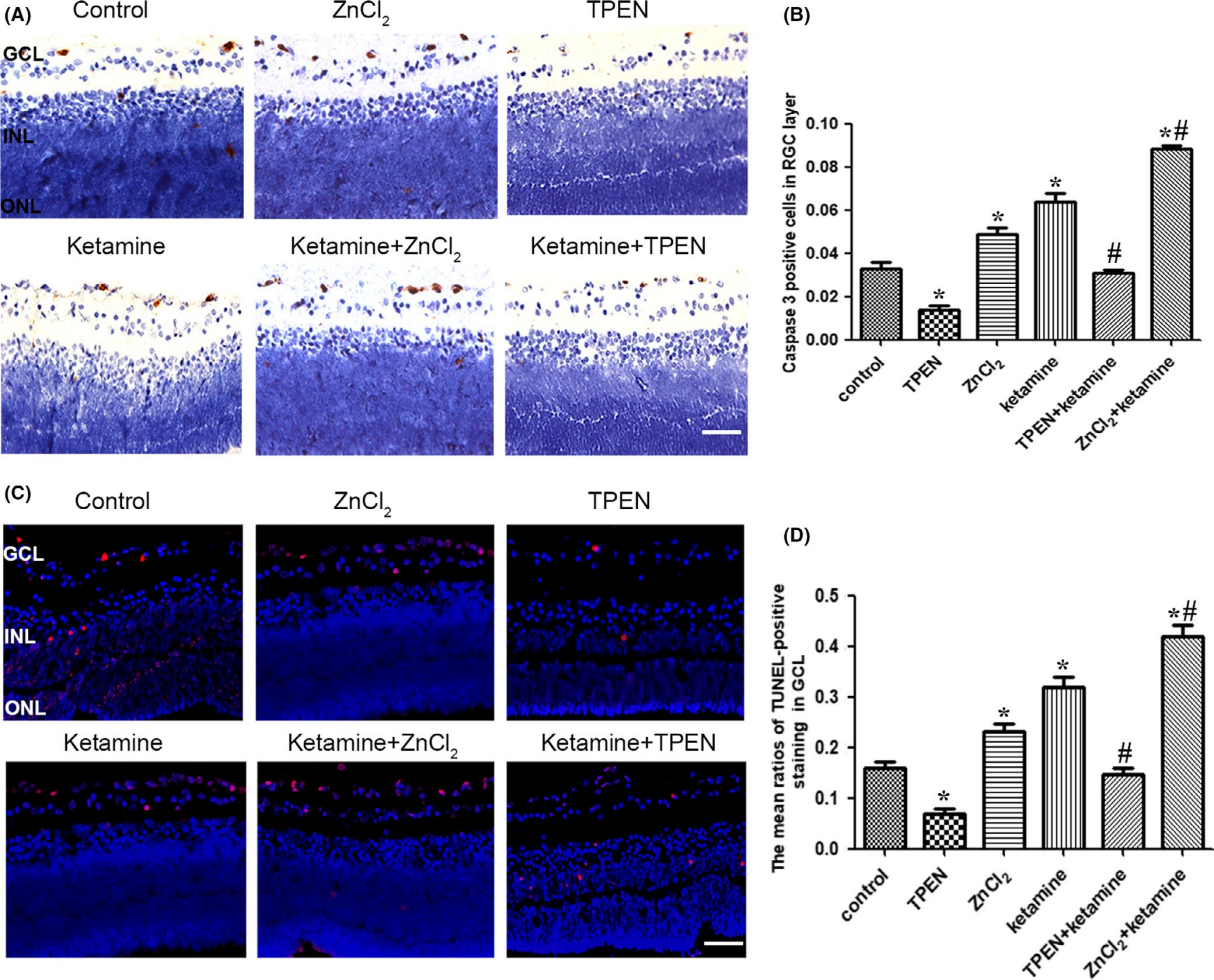
Treatment with a Zn^2+^ chelator attenuated ketamine‐induced RGC apoptosis in P7 rats. A, Representative photomicrograph of caspase‐3‐positive staining (brown) in the GCL (scale bar = 25 μm). B, Ratios of caspase‐3‐positive cells in the GCL. C, Representative photomicrograph of TUNEL‐positive staining (red) in the GCL (scale bar = 25 μm). D, Ratios of TUNEL‐positive cells in the GCL. GCL: ganglion cell layer; INL: inner nuclear layer; ONL: outer nuclear layer; P7: postnatal day 7; RGC: retinal ganglion cell; TUNEL: terminal deoxynucleotidyl transferase biotin‐dUTP nick end labeling. *P < .05 relative to the control group; #*P* < .05 relative to the ketamine group

The immunohistochemistry and TUNEL assays revealed that exposure to 100 μmol/L TPEN significantly decreased the extent of neuronal apoptosis in the GCL of the rat retina (Figure 6). The percentage of AC3‐positive neurons decreased from 3.2 ± 1.3% to 1.4 ± 0.9% (*P* < .001, n = 5), and the percentage of TUNEL‐positive cells decreased from 15.8 ± 7.2% to 7.0 ± 4.8% (*P* = .001, n = 5). Conversely, exposure to 100 μmol/L ZnCl_2_ increased the percentage of AC3‐positive neurons from 3.2 ± 1.3% to 4.9 ± 1.3% (*P* = .006, n = 5) and the percentage of TUNEL‐positive cells from 15.9 ± 7.1% to 23.0 ± 8.4% (*P* = .004, n = 5).

To investigate the effect of Zn^2+^ on ketamine‐induced apoptosis, we further investigate whether TPEN and ZnCl_2_ affected ketamine‐induced neuronal apoptosis. Immunohistochemistry and TUNEL assays also revealed that 100 μmol/L TPEN treatment decreased the extent of ketamine‐induced neuronal apoptosis in the GCL. The percentage of AC3‐positive neurons decreased from 6.4 ± 2.2% to 3.1 ± 0.7% (*P* < .001, n = 5), and the percentage of TUNEL‐positive cells decreased from 31.8 ± 10.9% to 14.6 ± 6.9% (*P* < .001, n = 5). Exposure to 100 μmol/L ZnCl_2_ increased the extent of ketamine‐induced neuronal apoptosis in the GCL. The percentage of AC3‐positive neurons increased from 6.4 ± 2.2% to 8.8 ± 0.9% (*P* < .001, n = 5), and the percentage of TUNEL‐positive cells increased from 31.8 ± 10.9% to 41.9 ± 10.8% (*P* < .001, n = 5).

## 4 DISCUSSION

Our study demonstrated that long‐term ketamine exposure was able to induce neuronal apoptosis in early developing rat retina. Blockade of the non‐integrin laminin receptor promoted ketamine‐induced neuronal apoptosis, while non‐integrin laminin receptor activation attenuated ketamine‐induced apoptotic responses. Ketamine‐induced laminin degradation, possibly through enhancing the activity of MMP9. Inhibition of MMP9 activity ameliorated ketamine‐induced neuronal apoptosis through reducing laminin degradation. Furthermore, we also found that downregulation of Zn^2+^played a protective role against ketamine‐induced neuronal apoptosis through inhibiting MMP9 activity and laminin degradation.

A previous study suggested that laminin degradation induced neuronal apoptosis in the newborn hippocampus.^16^ Furthermore, siRNA‐mediated downregulation of the non‐integrin LR induces apoptosis and reduces cellular viability in different cancer cells.^22^, ^31^ Our study found that laminin was degraded after the administration of ketamine; blockade of the non‐integrin LR promoted ketamine‐induced neuronal apoptosis, while activation of the non‐integrin LR attenuated apoptosis. This suggests that laminin degradation is involved in ketamine‐induced apoptosis, possibly through a non‐integrin receptor‐mediated pathway. The non‐integrin LR plays important roles in cell migration, invasion, angiogenesis, ECM remodeling, and apoptosis.^22^, ^32^, ^33^ However, laminin‐integrin binding has also been reported to modulate neuronal survival through the Akt or focal adhesion kinase signaling pathway.^16^, ^34^ Whether the laminin‐integrin signaling pathway is involved in ketamine‐induced apoptosis needs further investigation.

Previous studies have demonstrated that laminin can be degraded by MMPs, including MMP9, during brain development.^16^, ^28^ Our study found that the MMP9 inhibitor increased laminin expression in developing neuron, which is consistent with the previous finding. Furthermore, MMP9 inhibition also ameliorated ketamine‐induced laminin degradation. This result suggests that reducing laminin degradation via MMP9 inhibition may attenuate neuronal apoptosis. Previous studies have reported that MMP9 inhibition enhances cell survival in various models.^35^, ^36^ We further confirmed that administration of MMP9 inhibitor reduced physiological and ketamine‐induced apoptosis in the GCL at P7, suggesting that ketamine‐induced neuronal apoptosis may be associated with increased MMP9 activity.

Previous studies have demonstrated that MMP9 is upregulated and leads to neuronal cell death after various insults.^36^, ^37^, ^38^ Our study found that the general anesthetic ketamine increased MMP9 expression and activity. Similarly, a previous study showed that the enzymatic activity of MMP2 increased after treatment with the non‐competitive NMDA receptor antagonist MK801 in newborn rats.^39^ In contrast, Uckermann et al did not detect any changes in MMP2 mRNA or protein expression in the immature brain after treatment with MK801.^40^ This difference can be explained by a different concentration of MK801 in the experimental protocol and the use of a different protein concentration in the MMP2 activity analysis in the latter study.^39^ Taken together, the ketamine‐induced laminin degradation observed in our study may be due to the enhanced activity of MMP9.

Previous studies have shown that dysregulation of Zn^2+^ can affect the survival and degeneration of neurons.^41^, ^42^ Liu et al found that the application of Zn^2+^ chelator TPEN can reduce PC12 cells death through glutamate signaling pathway and voltage‐dependent outward potassium current changes in oxygen and glucose deprivation model.^43^ In addition, Cho et al reported that subcutaneous injection of TPEN significantly reduced neuronal apoptosis in the brain by interfering with caspase‐dependent apoptosis pathways in P7 rats.^44^ Consistent with these studies, we found that downregulation of Zn^2+^ by TPEN attenuated ketamine‐induced neuronal apoptosis, while upregulation of Zn^2+^ increased neuronal apoptosis in the GCL of rat retina. However, we suggested that the mechanism that Zn^2+^ mediated neuronal apoptosis is related to the activity of MMP9. In our study, TPEN attenuated the increase of MMP9 activity induced by ketamine in the developing rat retina, while the upregulation of Zn^2+^ enhanced the activity of MMP9. Previous study has also shown that upregulation of Zn^2+^ can increase the activity of MMP9 in retinal detachment models.^45^ In addition, Hwang et al reported that upregulation of Zn^2+^ increased the activity of MMP9 in the mouse brain.^46^ Furthermore, our research found that upregulation of Zn^2+^ aggravated ketamine‐induced laminin degradation, and TPEN reduced the laminin degradation. The protective mechanism of TPEN in neuronal apoptosis may be related to that the decreased MMP9 activity leads to reduced degradation of laminin. Thus, it may be possible to reduce neurotoxicity caused by general anesthesia in children by modulating Zn^2+^to change the activity of MMP9 and laminin expression.

In the present study, the newborn rat retinas were used to examine the potential roles of zinc and MMP9 in neuronal apoptosis during early postnatal development. As an extension of the central nervous system, the retina is composed of three neuronal layers and provides an excellent model for the assessment of neuronal degeneration.^6^ Furthermore, the use of newborn rat retinas overcomes limitations associated with in vivo animal experiments, namely the effects of hypoxia and CO_2_ retention due to general anesthesia and physiological consequences of stress/physiological responses to stress.

With respect to the limitations of this study, we focused on cell counts to the GCL, but there are Caspase 3 + and TUNEL + cells present in places other than the GCL. The concentration of Zn^2+^ in the retina was not detected after the administration of ZnCl_2_ and TPEN. The mechanism that ketamine increased MMP9 expression and activity in early development needs further study. Furthermore, we did not study the role of integrin in laminin degradation‐induced apoptosis. Therefore, the roles of LRs and other signaling pathways in ketamine‐induced neuronal apoptosis during early development require further exploration.

## 5 CONCLUSION

In conclusion, this study showed that laminin degradation by MMP9 promoted ketamine‐induced neuronal apoptosis in early developing rat retina. The non‐integrin LR may be a pathway involved in ketamine‐induced apoptosis. Zn^2+^ downregulation may play a protective role against ketamine‐induced neuronal apoptosis through inhibiting MMP9 activation and laminin degradation.

## CONFLICT OF INTEREST

The authors declare no conflict of interest.

## Supporting information

Supplementary Material
