# Supplementary material for: Laminin degradation by matrix metalloproteinase 9 promotes ketamine‐induced neuronal apoptosis in the early developing rat retina
Source: CNS Neurosci Ther. 2020 Jun 20;26(10):1058–68. doi: 10.1111/cns.13428 (PMC7539835; doi:10.1111/cns.13428)
Supplement: Supplementary file 1 — Supplementary Material [file CNS-26-1058-s001.pdf]

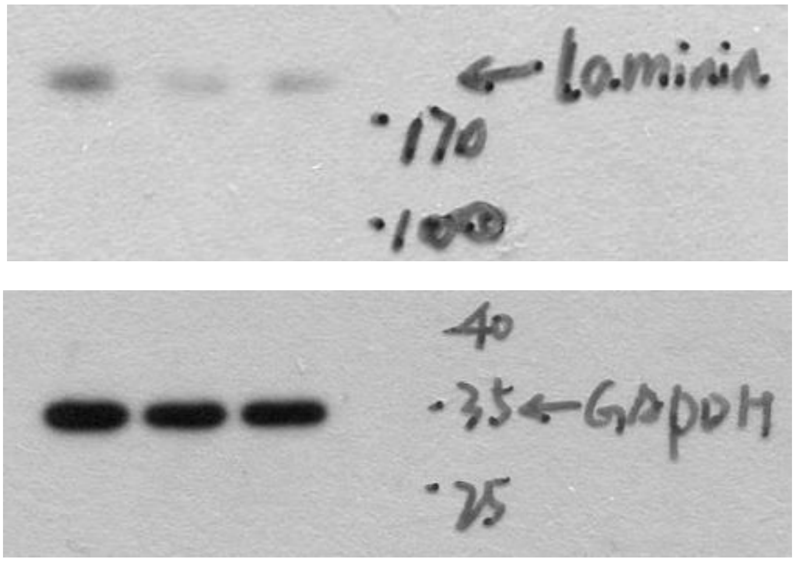

Full unedited gel/blot for Figure 2

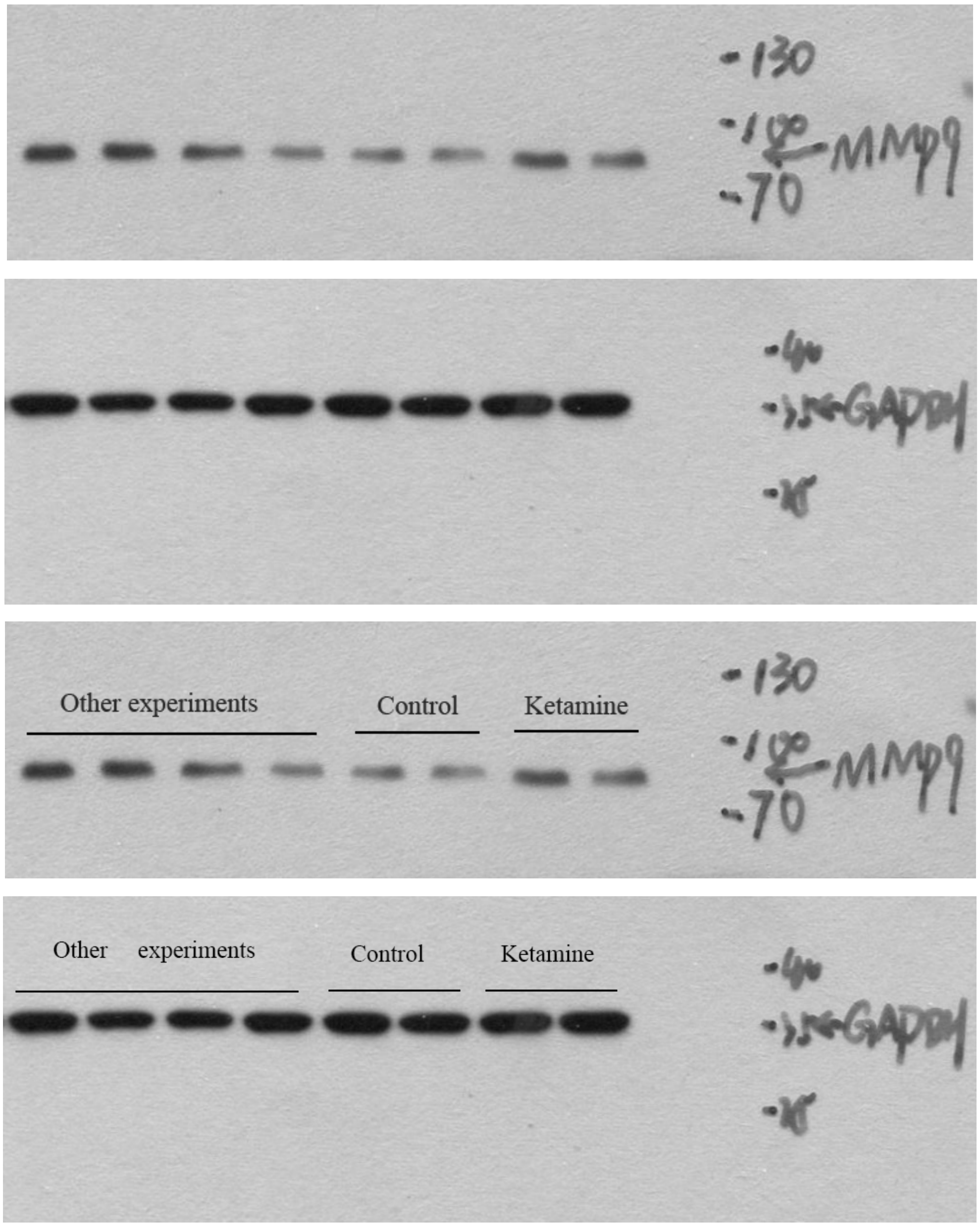

Full unedited gel/blot for Figure 4

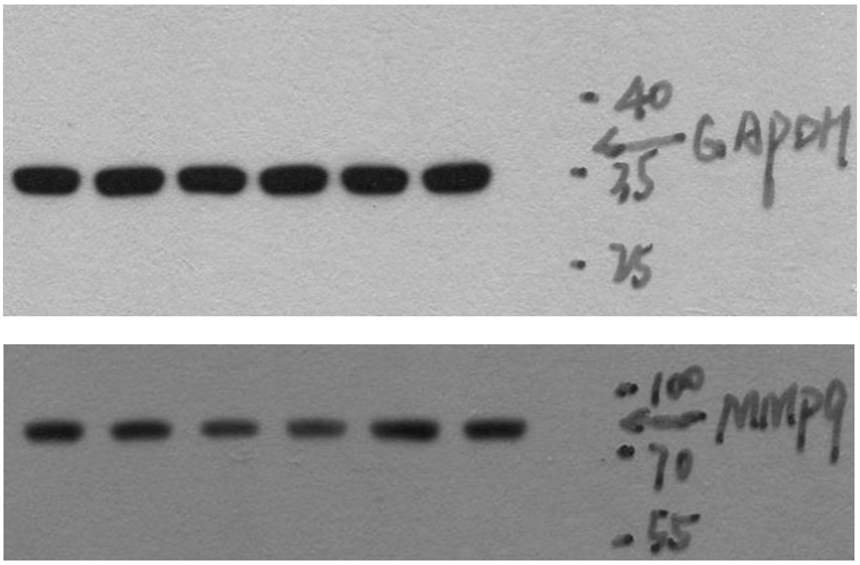

Full unedited gel/blot for Figure 5
